# EEG emotion recognition based on cross-frequency granger causality feature extraction and fusion in the left and right hemispheres

**DOI:** 10.3389/fnins.2022.974673

**Published:** 2022-09-07

**Authors:** Jing Zhang, Xueying Zhang, Guijun Chen, Lixia Huang, Ying Sun

**Affiliations:** College of Information and Computer, Taiyuan University of Technology, Taiyuan, China

**Keywords:** electroencephalogram, emotion recognition, Granger causality (GC), cross-frequency analysis, feature extraction, multi-feature fusion

## Abstract

EEG emotion recognition based on Granger causality (GC) brain networks mainly focus on the EEG signal from the same-frequency bands, however, there are still some causality relationships between EEG signals in the cross-frequency bands. Considering the functional asymmetric of the left and right hemispheres to emotional response, this paper proposes an EEG emotion recognition scheme based on cross-frequency GC feature extraction and fusion in the left and right hemispheres. Firstly, we calculate the GC relationship of EEG signals according to the frequencies and hemispheres, and mainly focus on the causality of the cross-frequency EEG signals in left and right hemispheres. Then, to remove the redundant connections of the GC brain network, an adaptive two-stage decorrelation feature extraction scheme is proposed under the condition of maintaining the best emotion recognition performance. Finally, a multi-GC feature fusion scheme is designed to balance the recognition accuracy and feature number of each GC feature, which comprehensively considers the influence of the recognition accuracy and computational complexity. Experimental results on the DEAP emotion dataset show that the proposed scheme can achieve an average accuracy of 84.91% for four classifications, which improved the classification accuracy by up to 8.43% compared with that of the traditional same-frequency band GC features.

## 1. Introduction

Electroencephalogram (EEG) is a kind of physiological electrical signal that can reflect dynamic changes of the central nervous system. In recent years, EEG signals have been widely used in emotion recognition because of their more objective response to the emotional state (Alarcao and Fonseca, 2017; Esposito et al., 2020; Rahman et al., 2021; Wu et al., 2022).

Modern cognitive neuroscience has indicated that the brain hemispheres are anatomically and functionally asymmetric (Dimond et al., 1976; Zheng and Lu, 2015; Li et al., 2018; Cui et al., 2020). In Zheng and Lu (2015), the cognitive differences between the left and right hemispheres have been revealed by analyzing the emotional cognitive characteristics induced by emotion stimulation, and found that the right hemisphere has more power in perceiving negative emotion. In Li et al. (2018), the differential entropy of each pair of EEG channels at the symmetrical position of the two hemispheres was calculated and used to obtain the differential asymmetry (DASM) and rational asymmetry (RASM), which has been proved to be effective in distinguishing emotional states. In Cui et al. (2020), a bi-hemisphere domain adversarial neural network model was designed to effectively improve the performance of EEG emotion recognition. Therefore, analyzing the EEG signals of the left and right hemispheres is of great significance for improving emotional recognition.

EEG brain network is one of the most effective methods for analyzing EEG signals, where each EEG channel represents a node and the connections between nodes are defined as the edges. According to whether the edges may be directed or not, brain connectivity can be subdivided into functional connectivity and effective connectivity (Jiang et al., 2004; Li et al., 2019; Cao et al., 2020, 2022). Functional connectivity is defined as statistical interdependence among the EEG signals, while effective connectivity can further measure the causal relationships of EEG signals. Granger causality (GC) (Granger, 1969) is an effective connection measure and the GC brain network has been extensively used to explore the causality of EEG signals in recent years (Dimitriadis et al., 2016; Tian et al., 2017; Jiang et al., 2019; Gao et al., 2020; Li T. et al., 2020; Chen et al., 2021). Generally, the EEG signal is usually decomposed into four bands: θ, α, β, and γ bands to define the change in brain state. In Gao et al. (2020), the GC brain network was constructed for β-band EEG signals to analyze the difference between calm and stress emotion states. In Dimitriadis et al. (2016), by calculating the cross-frequency causal interaction between the EEG signals of θ~α bands under a mental arithmetic task, the mechanism of human brain processing was deeply analyzed. However, the causal analysis in the current studies either within the same-frequency bands (e.g., β band) or via specific cross-frequency interactions (e.g., θ~α) (Yeh et al., 2016), and the effect of the cross-frequency causal interaction between EEG signals for on the emotion recognition is not completely analyzed. In essence, there is a causal relationship between EEG signals both in same-frequency and cross-frequency bands. Thus, it is necessary to carry out causality analysis among the EEG signal with different frequency bands and different hemispheres for emotion recognition.

On the other hand, it is a key issue to extract effective features from the GC brain network. The existing researches always selects the empirical threshold to directly converted the GC adjacency matrix into a binary data, that is, the GC values below the threshold are set to 0, and the GC values above the threshold value are set to 1. Then, the binary matrix is converted to network attributes as brain cognitive features based on the graph theory (De Vico Fallani et al., 2017; Hu et al., 2019; Covantes-Osuna et al., 2021; Li et al., 2022). In this way, the weak connections with lower GC values may be lost for the values below the threshold are set to 0. Otherwise, De Vico Fallani et al. (2012) have pointed out that the brain network exhibits a natural high redundant connection in all frequency bands, but the threshold method cannot effectively remove these redundant connections. Instead of the threshold method, this paper proposed an adaptive two-stage decorrelation (ATD) feature extraction method to improve the performance of emotion recognition, in which the redundant GC connections are adaptively removed under the goal of maintaining the optimal emotion recognition performance.

As is well-known from previous studies, a single type of GC feature can only show a part of causality information. In order to describe the causal interaction during emotion response more accurately, it is necessary to integrate the same-frequency GC features and cross-frequency GC features. Previous works on feature fusion aim to directly combine different types of feature vectors by concatenation, parallel or weighted fusion to improve the recognition accuracy (Yang et al., 2019; Bota et al., 2020; Cai et al., 2020; Li Y. et al., 2020; Yilmaz and Kose, 2021; An et al., 2022). However, existing feature fusion methods do not consider the increase in computational cost caused by the increase in feature numbers. Based on this, this paper designs a new multi-GC feature fusion scheme, which can effectively improve the performance of the EEG emotion recognition without increasing the number of features.

As mentioned above, this paper mainly studies the EEG emotion recognition based on cross-frequency GC feature extraction and fusion in the left and right hemispheres. Our contributions mainly focus on the following aspects: Firstly, the EEG electrode channels are divided into the left and right hemispheres according to their spatial position, and the EEG signals are decomposed into θ, α, β, and γ bands. The corresponding GC adjacency matrices are then constructed to analyze the causality of the EEG signals. Secondly, based on the characteristics of GC adjacency matrix, an ATD method is further proposed to remove the redundant connections in the GC brain network and extract the causal features for emotion recognition. Finally, a new weighted feature fusion scheme is designed that takes into account the emotion recognition accuracy and the feature number of each single GC feature, which can effectively improve the performance of the EEG emotion recognition system without increasing the computational cost. Experimental results of arousal-valence classification on the DEAP emotion dataset (Koelstra et al., 2011) show that the proposed scheme can achieve an average recognition improvement of 8.43% than that of the same-frequency GC features.

The remaining parts are organized as follows. Section 2 reviews the DEAP emotion dataset and GC theory. Section 3 describes the proposed EEG emotion recognition method, including the frequency-hemisphere GC measure for the EEG signal, the ATD feature extraction method, and the proposed multi-feature fusion scheme. Section 4 presents the experimental results and discussions. Finally, some conclusions are presented in Section 5.

## 2. Related works

### 2.1. DEAP emotion dataset

The DEAP dataset (Koelstra et al., 2011) is a multimodal dataset for analyzing human affective states, which consists of EEG signals and peripheral physiological signals of 32 subjects. During the experiment, all subjects watched 40 excerpts of one-minute music videos. At the end of each trial, participants performed a self-assessment of their levels of arousal, valence, linking, and dominance using SAM mannequins on a discrete 9-point scale. The arousal scale ranged from calm to excited, the valence scale ranged from unhappy to happy, the linking scale measured the personal preferences of the participants for a given media, and the dominance scale ranged from submissive to dominant. The EEG signals were recorded from 32 channels according to the international 10–20 system, at a sampling rate of 512 Hz, while peripheral physiological signals including skin temperature, blood volume pressure, an electromyogram, and galvanic skin response, were recorded from another 8 channels.

In this paper, only the EEG signal is used to investigate EEG emotion recognition research. According to the 1 9 self-assessment scores of participants, we select the median score 5 as the threshold, with higher than 5 representing high class and less than or equal to 5 representing low class. The valence-arousal (VA) space is divided into four parts, i.e., low arousal-low valence (LALV), high arousal-low valence (HALV), low arousal-high valence (LAHV), and high arousal-high valence (HAHV).

### 2.2. Preprocessing and frequency band division of EEG signal

**Figure 1:**
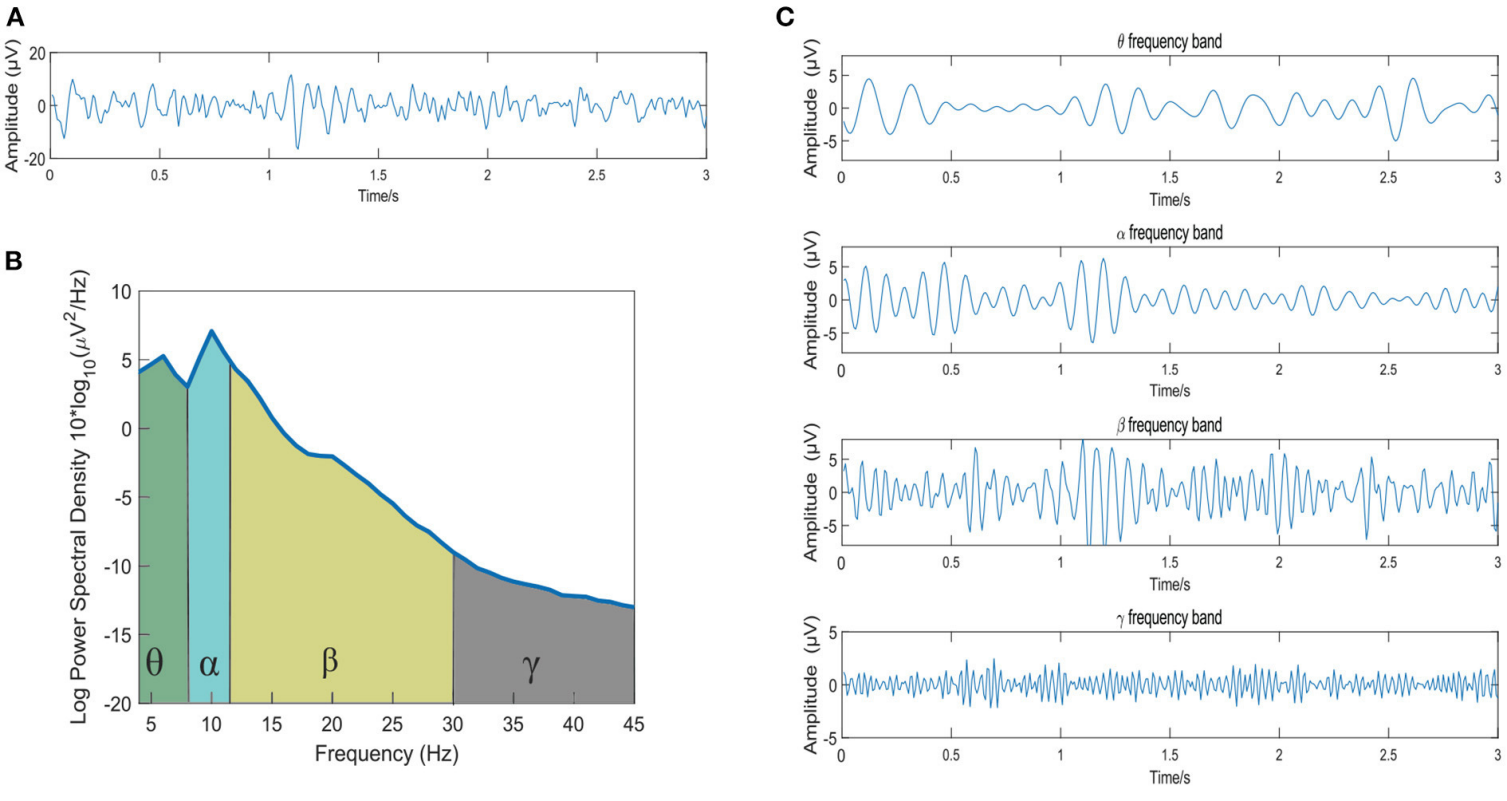
The frequency band division of a 3s EEG signal. **(A)** The original EEG signal; **(B)** The power spectral density of the EEG signal; **(C)** The EEG signals of the θ, α, β, and γ frequency bands extracted by STFT method.

The original raw EEG data is first carried out in the following 4 preprocessing steps: (1) down-sampling to 128 Hz, (2) removal of the EOG artifacts, (3) bandpass filtering of the raw data between 4 and 45 Hz, and (4) averaging of the data to a common reference. Then, the Short-time Fourier Transform (STFT) is then used to extract the θ (4~8 Hz), α (8~12 Hz), β (12~30 Hz) and γ (30~45 Hz) bands. To observe the waveform of EEG signal decomposed into four frequency bands more intuitively, Figure 1 shows the frequency band division of a 3s EEG signal in the DEAP dataset. Figure 1A shows the original EEG signal, Figure 1B is the corresponding power spectral density of the EEG signal, and Figure 1C presents the four-band EEG signals extracted by STFT.

### 2.3. Overview of GC analysis

Granger causality is one of the most popular approaches for quantifying causal relationships between time series data, which introduced first in econometrics by Granger (1969). GC analysis is widely used in emotion recognition because of its strong interpretability. It is based on two major principles: (i) the cause happens prior to the effect, and (ii) the cause makes notable changes in the effect. Generally speaking, a time series *X* is said to “Granger cause" another time series *Y*, denoted by *X*→*Y*. More specifically, granger causality occurs if and only if the prediction values of *Y* based on the past values of *X* and *Y* are better than predictions based on the past values of *Y* alone. For two time series *X* and *Y*, a univariate and a bivariate vector autoregressive (VAR) model are performed to the predicted current values by the following regressions:


(1) $$\begin{array}{lll} X\left(t\right) & = & \overset{L}{\underset{i=1}{\sum }}a_{1i}X\left(t-i\right)+\varepsilon_{X} \end{array}$$

(2) $$\begin{array}{lll} Y\left(t\right) & = & \overset{L}{\underset{i=1}{\sum }}b_{1i}Y\left(t-i\right)+\varepsilon_{Y} \end{array}$$

(3) $$\begin{array}{lll} X\left(t\right) & = & \overset{L}{\underset{i=1}{\sum }}a_{2i}X\left(t-i\right)+\overset{L}{\underset{i=1}{\sum }}b_{2i}Y\left(t-i\right)+\eta_{YX} \end{array}$$

(4) $$\begin{array}{lll} Y\left(t\right) & = & \overset{L}{\underset{i=1}{\sum }}c_{2i}X\left(t-i\right)+\overset{L}{\underset{i=1}{\sum }}d_{2i}Y\left(t-i\right)+\eta_{XY} \end{array}$$

where *a*_1*i*_, *b*_1*i*_, *a*_2*i*_, *b*_2*i*_, *c*_2*i*_, and *d*_2*i*_(*i* = 1, 2, …, *L*) are the constant coefficients, and *L* is the order of the model, both of them can be obtained through the Bayesian information criterion. ε_*X*_ and ε_*Y*_ represent the error of the univariate model, η_*YX*_ and η_*XY*_ represent the error of the bivariate model, respectively.

The mathematical definition of GC is the logarithm of the two ratios of the error variances: the variance of the errors from the univariate VAR model and bivariate VAR model.


(5) $$\begin{array}{lll} F_{X\to Y} & = & \ln\;\left(\frac{\sigma_{\varepsilon_{Y}}}{\sigma_{\eta_{XY}}}\right) \end{array}$$

(6) $$\begin{array}{lll} F_{Y\to X} & = & \ln\;\left(\frac{\sigma_{\varepsilon_{X}}}{\sigma_{\eta_{YX}}}\right) \end{array}$$

where the σ_ε_*X*__, σ_ε_*Y*__, σ_η_*XY*__, and σ_η_*YX*__ represent the variances of the error in Equations (1)–(4), respectively. When σ_ε_*X*__ is large than σ_η_*YX*__, means that *Y* is the “Granger Cause" to *X*. Similarly, when σ_ε_*Y*__ is large than σ_η_*XY*__, means that *X* is the “Granger Cause" to *Y*.

## 3. The proposed EEG emotion recognition scheme

**Figure 2:**
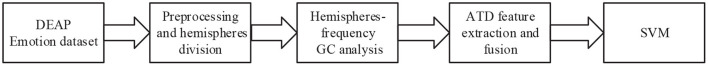
The framework of the proposed emotion recognition method.

Figure 2 shows the framework of the proposed EEG emotion recognition method. The preprocessing and hemispheres division module is first used to divide the original EEG signal into the left and right hemispheres according to their positions, and STFT is then used to extract the θ (4~8 Hz), α (8~12 Hz), β (12~30 Hz) and γ (30~45 Hz) bands. Hemispheres-frequency GC analysis module is then used to analyze the GC relationship of the EEG signals with different frequencies in the left and right hemispheres, and corresponding GC value is calculated to construct the adjacency matrix. After this, the ATD feature extraction and fusion module is adopted to adaptively remove the redundant connections in the GC brain network and extract the optimized GC features (GC+ATD), and design a multi-feature fusion scheme to integrate different GC+ATD features. Finally, the support vector machine (SVM) (Chang and Lin, 2011) classifier is used to obtain the emotion recognition results.

### 3.1. The proposed GC measures of left and right hemispheres

#### 3.1.1. The classification of hemispheres-frequency GC measures

**Figure 3:**
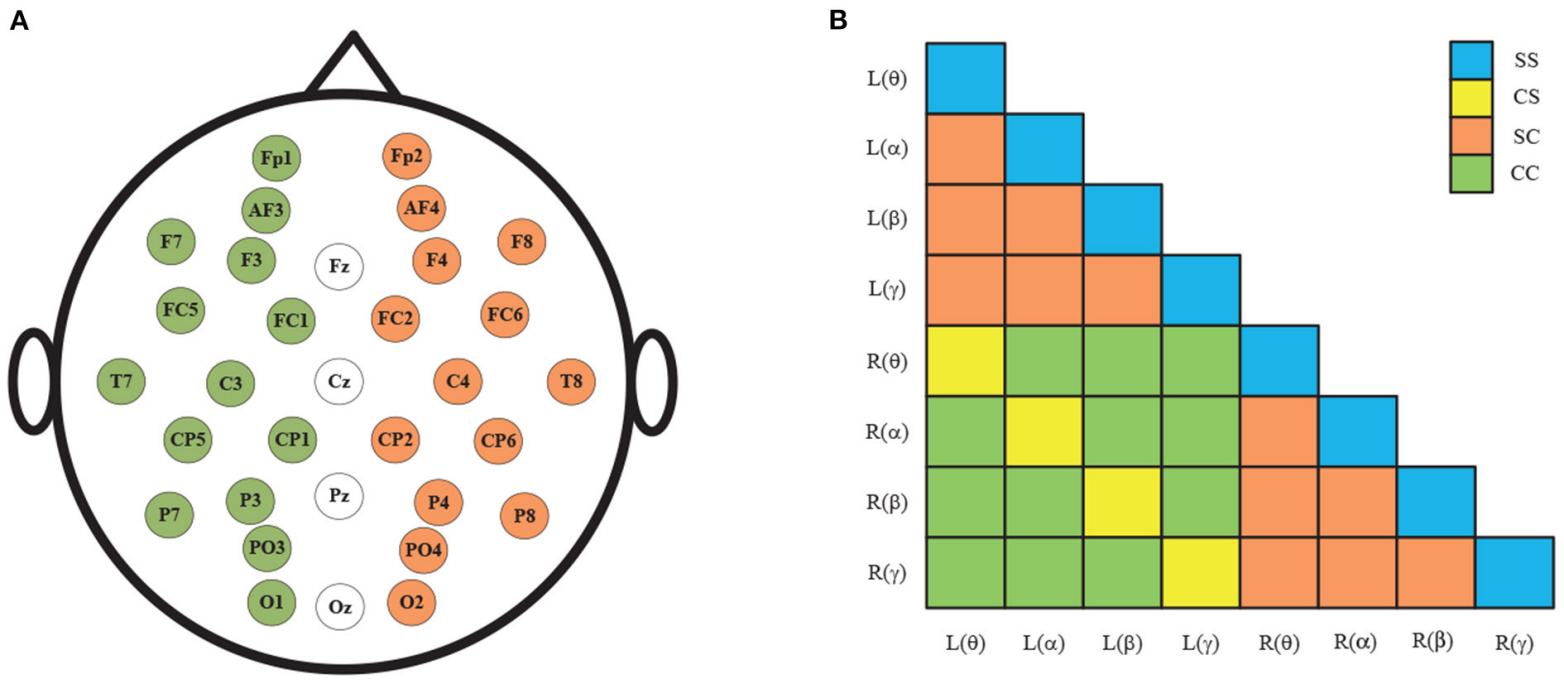
**(A)** 32 electrode positions in the international 10–20 system; **(B)** The 36 pairs GC combinations.

Figure 3A shows the spatial distribution of the 32 EEG electrodes in the international 10–20 system. Taking the middle four EEG electrodes as the axis, the human brain is divided into left and right hemispheres, with 14 EEG electrodes in each hemisphere. After the removal of four central electrodes (*Fz*, *Cz*, *Pz*, and *Pz*), the remaining electrodes are symmetrical and divided into 14 pairs of left and right combinations: *Fp*1-*Fp*2, *AF*3-*AF*4, *F*7-*F*8, *F*3-*F*4, *FC*5-*FC*6, *FC*1-*FC*6, *T*7-*T*8, *C*3-*C*4, *CP*5-*CP*6, *CP*1-*CP*2, *P*7-*P*8, *P*3-*P*4, *PO*3-*PO*4, and *O*1-*O*2. Finally, the hemispheres causality and frequencies causality of EEG signals is combined and resulting in 36 combinations as shown in Figure 3B.

As shown in Figure 3B, the GC combinations can be furtherly divided into four categories according to whether the hemispheres and frequencies are the same: (1) same-hemisphere and same-frequency (SS), which reflects the GC of EEG signals with the same hemispheres and same frequency domain; (2) cross-hemisphere and same-frequency (CS), which reflects the GC of the same-frequency EEG signals between the left and right hemispheres; (3) same-hemisphere and cross-frequency (SC), which reflects the GC of cross-frequency EEG signals with the same hemisphere; (4) cross-hemisphere and cross-frequency (CC), which reflects the GC of cross-frequency EEG signals between the left and right hemispheres.

**Figure 4:**
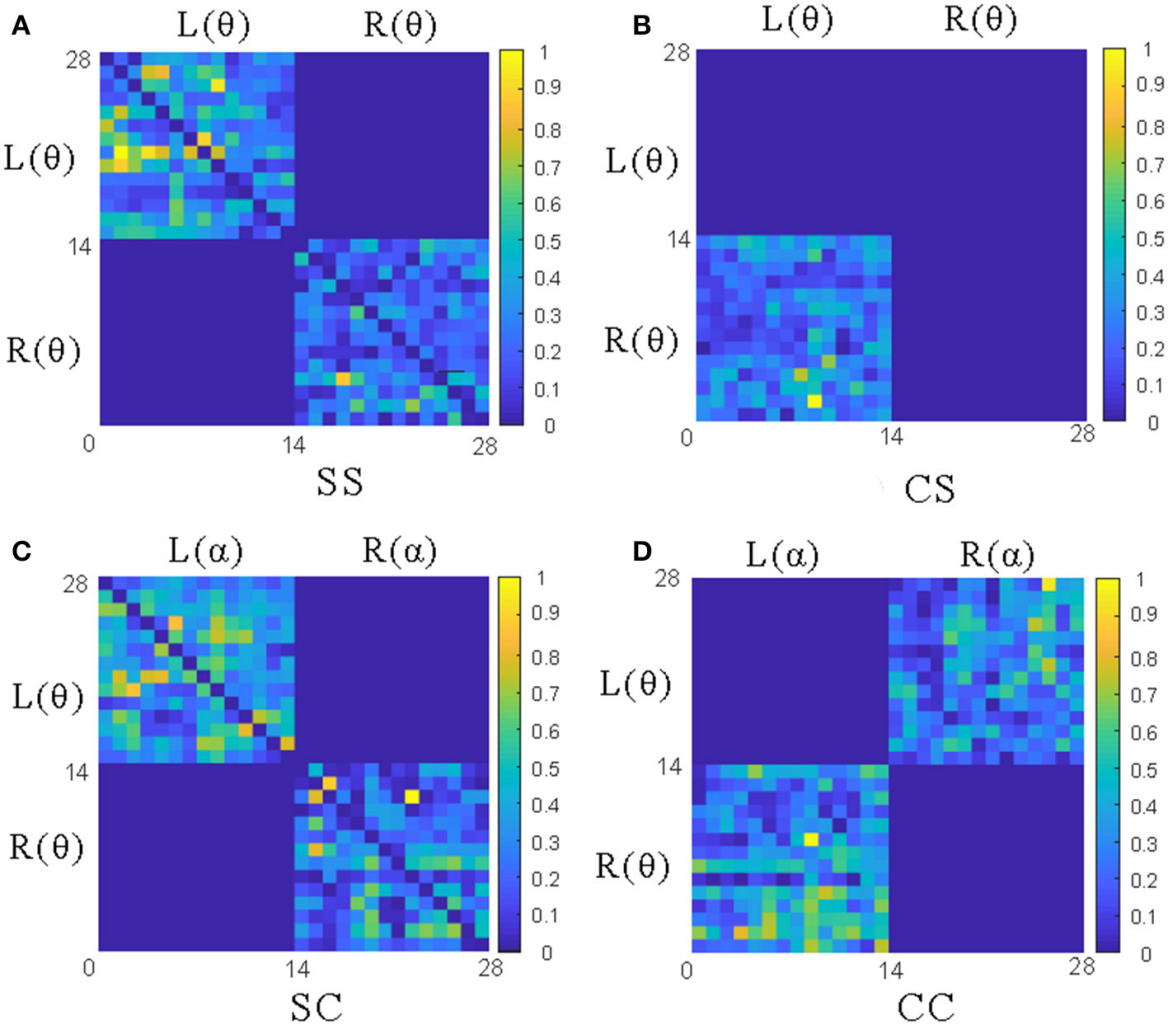
The adjacency matrices of four GC measures between the θ band and the θ~α band. **(A)** The L(θ)~L(θ) and R(θ)~R(θ) matrices of SS measures; **(B)** The L(θ)~ R(θ) matrix of CS measures; **(C)** The L(θ)~L(α)and R(θ)~R(α) matrices of CS measures; **(D)** The L(θ)~R(α) and L(α)~ R(θ) matrices of CC measures.

To analyze the above measures more specifically, the proposed GC adjacency matrices between the θ band and the θ~α band of an EEG signal are shown in Figure 4, where *L* and *R* represent the left and right hemispheres, and θ and α represent the frequency band. For example, *L*(θ) represents the θ-frequency EEG signals in the left hemisphere. Since there are 14 EEG electrodes in each hemisphere, the size of the corresponding GC matrix is 28*28. Among them, SS and CS belong to the same-frequency GC measures, which have been widely explored in the existing research. On the contrary, SC and CC belong to the cross-frequency GC measures, which are rarely involved in emotion recognition, especially CC measure. Therefore, we mainly focus on the analysis of the CC measure in this subsection, and the other three measures can be analyzed to CC.

As shown in Figure 3B, the CC measures are pairwise symmetric and can be combined into six groups: θ~α, θ~β, θ~γ, α~β, α~γ and β~γ. In the following, we will take the CC(θ~α) as an example to analyze the GC relationship of EEG signals. As shown in Figure 4D, the lower-left and the upper-right of the CC(θ~α) matrix are the values of *L*(α)~*R*(θ) and *L*(θ)~*R*(α), while the rest values (including the lower-right and the upper-left) of the same hemisphere are set to 0. After the removal of four central electrode nodes from the 32 electrode nodes, both of left and right hemispheres have 14 electrode nodes, and the number of effective GC values in the CC(θ~α) is 14*14*2=392. Similarly, the number of GC values in SS, CS and SC can be obtained, as shown in Table 1.

**Table 1:** The GC value numbers of different measures.

**GC measure**	**Frequency-hemispheres combination**	**The GC value numbers of single combination**	**The GC value numbers of all combinations**
SS	θ~θ, α~α, β~β, γ~γ	392	1,568
CS	θ~θ, α~α, β~β, γ~γ	196	784
SC	θ~α, θ~β, θ~γ, α~β, α~γ, β~γ	392	2,352
CC	θ~α, θ~β, θ~γ, α~β, α~γ, β~γ	392	2,352

#### 3.1.2. The discussion of CC measure

**Figure 5:**
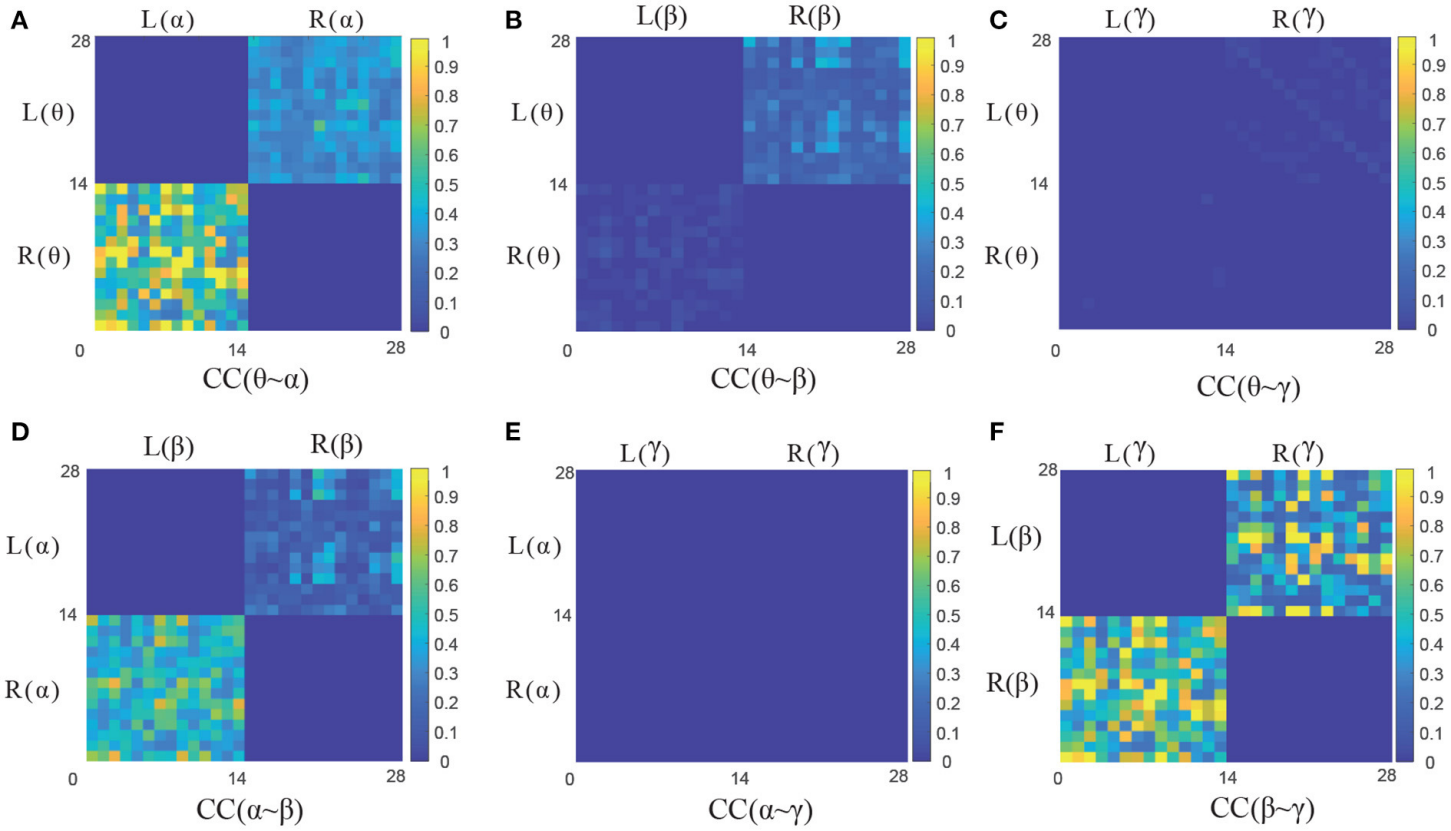
The GC adjacency matrices of six CC measures. **(A–F)** Illustrate the GC adjacency matrix of CC(θ~α), CC(θ~β), CC (θ~γ), CC(α~β), CC(α~γ), and CC(β~γ), respectively.

To further analyze the characteristics of six frequency-hemispheres combinations in CC causal measurement, the corresponding the GC adjacency matrix of CC(θ~α), CC(θ~β), CC(θ~γ), CC(α~β), CC(α~γ) and CC(β~γ) are presented in Figures 5A–F, respectively. It can be seen that there is a significant GC relationship between EEG signals in the adjacent frequencies, such as CC(θ~α), CC(α~β), and CC(β~γ), where CC(β~γ) holds the strongest causality. On the contrary, there is almost no causality between EEG signals in the non-adjacent frequencies, such as CC(θ~β), CC(θ~γ) and CC(α~γ). Therefore, the causal features of the adjacent frequency EEG signals can be effectively used for improving the emotion recognition performance, and how to extract the causal features from the GC adjacency matrix will be discussed in detail in the following section.

#### 3.1.3. Formula derivation of CC(θ~α) measure

In order to extract the features of the GC adjacency matrix, it is necessary to calculate the GC value. In this section, we will take CC(θ~α) measure as an example to describe the detailed calculation process. Let *X*_*L*(θ)_(*t*) and *Y*_*R*(α)_(*t*) denote the *t*-th time-lagged values of *L*(θ) and *R*(α), respectively. Similar to Equations (1)–(4), the univariate GC model and the bivariate GC model of *X*_*L*(θ)_(*t*) and *Y*_*R*(α)_(*t*) can be expressed as follows:


(7) $$\begin{array}{lll} X_{L\left(\theta \right)}\left(t\right) & = & \overset{L}{\underset{i=1}{\sum }}a_{1i}X_{L\left(\theta \right)}\left(t-i\right)+\varepsilon_{X} \end{array}$$

(8) $$\begin{array}{lll} Y_{R\left(\alpha \right)}\left(t\right) & = & \overset{L}{\underset{i=1}{\sum }}b_{1i}Y_{R\left(\alpha \right)}\left(t-i\right)+\varepsilon_{Y} \end{array}$$

(9) $$\begin{array}{lll} X_{L\left(\theta \right)}\left(t\right) & = & \overset{L}{\underset{i=1}{\sum }}a_{2i}X_{L\left(\theta \right)}\left(t-i\right)+\overset{L}{\underset{i=1}{\sum }}b_{2i}Y_{R\left(\alpha \right)}\left(t-i\right)+\eta_{YX} \end{array}$$

(10) $$\begin{array}{lll} Y_{R\left(\alpha \right)}\left(t\right) & = & \overset{L}{\underset{i=1}{\sum }}c_{2i}X_{L\left(\theta \right)}\left(t-i\right)+\overset{L}{\underset{i=1}{\sum }}d_{2i}Y_{R\left(\alpha \right)}\left(t-i\right)+\eta_{XY} \end{array}$$

where *a*_1*i*_, *b*_1*i*_, *a*_2*i*_, *b*_2*i*_, *c*_2*i*_, and *d*_2*i*_(*i* = 1, 2, …, *L*) are the constant coefficients, and *L* is the order of the model.

Then, according to Equations (5) and (6), the GC values of *X*_*L*(θ)_→*Y*_R(α)_ and *Y*_R(α)_→*X*_*L*(θ)_ are


(11) $$\begin{array}{lll} F_{X_{L\left(\theta \right)}\to Y_{R\left(\alpha \right)}} & = & \ln\;\left(\frac{\sigma_{\varepsilon_{Y}}}{\sigma_{\eta_{XY}}}\right) \end{array}$$

(12) $$\begin{array}{lll} F_{Y_{{\;}_{R\left(\alpha \right)}}\to X_{L\left(\theta \right)}} & = & \ln\;\left(\frac{\sigma_{\varepsilon_{X}}}{\sigma_{\eta_{YX}}}\right) \end{array}$$

Since *F*_*X*_*L*(θ)_→*Y*_*R*(α)__ and *F*_*Y*__*R*(α)__→*X*_*L*(θ)__ represents the GC value the upper right and lower left of the *L*(θ)~*R*(α), respectively. Therefore, these two feature vectors are directly cascade as the GC values of *L*(θ)~*R*(α) and take *concat* to represent this process:


(13) $$\begin{array}{l} F_{L\left(\theta \right)\sim R\left(\alpha \right)}=concat\left(F_{X_{L\left(\theta \right)}\to Y_{R\left(\alpha \right)}},F_{Y_{{\;}_{R\left(\alpha \right)}}\to X_{L\left(\theta \right)}}\right) \end{array}$$

According to the Figures 3B, 4D, the *CC*(*L*(θ)~*R*(α)) and *CC*(*L*(α)~*R*(θ)) are symmetric. Therefore, *F*_CC(L(α)~R(θ))_ can be calculated similarly by Equations (7)–(13). As a result, the GC values of CC (θ~α) can be expressed as:


(14) $$\begin{array}{l} F_{CC\left(\theta \sim \alpha \right)}=concat\left(F_{CC\left(L\left(\theta \right)\sim R\left(\alpha \right)\right)},F_{CC\left(L\left(\alpha \right)\sim R\left(\theta \right)\right)}\right) \end{array}$$

### 3.2. The propose ATD method

**Figure 6:**
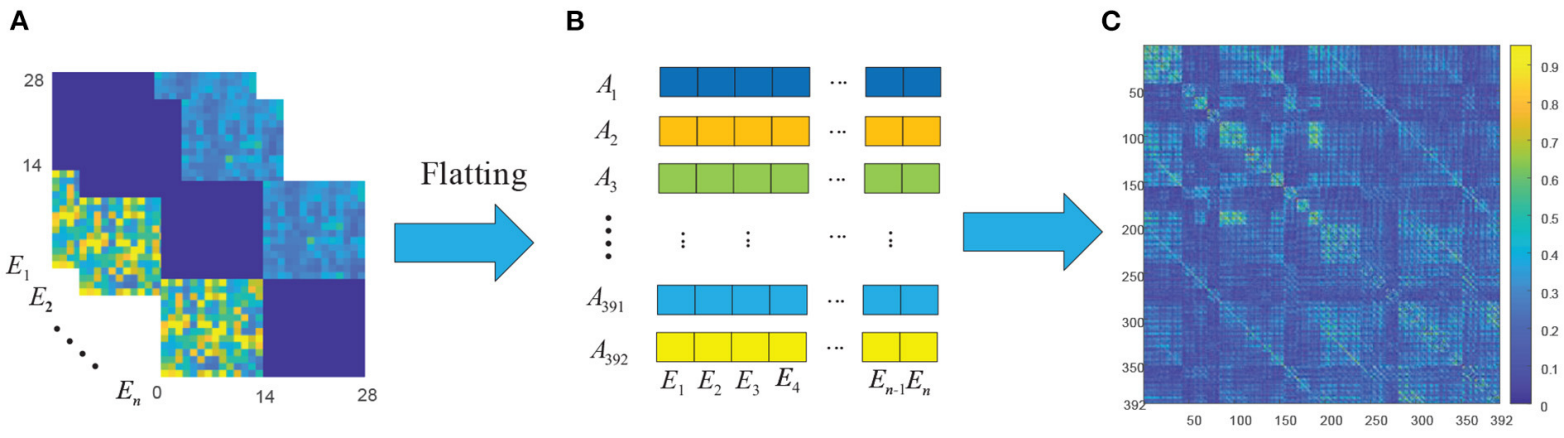
The correlation analysis of CC(θ~α) brain network. **(A)** The CC(θ~α) adjacency matrices of *n-segment* EEG signals; **(B)** 392-*dim* feature set F0 of *n-segment* EEG signals; **(C)** The correlation coefficient matrix of 392 features in F0.

Due to redundant connections in the GC brain network, this section takes CC(θ~α) as an example for detailed analysis of the ATD method. We select *n*-segment EEG signals in the DEAP dataset to construct the CC(θ~α) brain networks and calculate the corresponding adjacency matrices *E*_*i*_(*i* = 1, 2…*n*), as shown in Figure 6A. Table 1 shows that the number of GC values in each CC(θ~α) matrix is 392, and these 392 GC values can be calculated according to the calculation procession in Section 3.1.3. Then, the GC values of the same spatial position in CC adjacency matrices are constructed as a *n-dim* feature vector and resulting in 392 *n-dim* feature vectors *A*_*i*_(*i* = 1, 2…392), as shown in Figure 6B.

Next, we analyze the correlation between two feature vectors of *A*_*i*_(*i* = 1, 2…392). For any two features *A*_*u*_ and *A*_*v*_(*u, v* = 1, 2, …, 392, *u*≠*v*), the correlation coefficient *r*(*A*_*u*_, *A*_*v*_) between them can be calculated from Equation (15). The correlation coefficient matrix $R_{A}\in {\mathbb{R}}^{392\times 392}$ of 392 feature vectors can be obtained and shown in Figure 6C, where the value is closer to 1 with the greater correlation, and the value is closer to 0 with the smaller correlation.


(15) $$\begin{array}{l} r\left(A_{u}\left(i\right),A_{v}\left(i\right)\right)=\frac{\overset{n}{\underset{i=1}{\sum }}\left(A_{u}\left(i\right)-\bar{A_{u}}\right)\left(A_{v}\left(i\right)-\bar{A_{v}}\right)}{\sqrt{\overset{n}{\underset{i=1}{\sum }}{\left(A_{u}\left(i\right)-\bar{A_{u}\left(i\right)}\right)}^{2}}\sqrt{\overset{n}{\underset{i=1}{\sum }}{\left(A_{v}\left(i\right)-\bar{A_{v}}\right)}^{2}}} \end{array}$$

**Figure 7:**
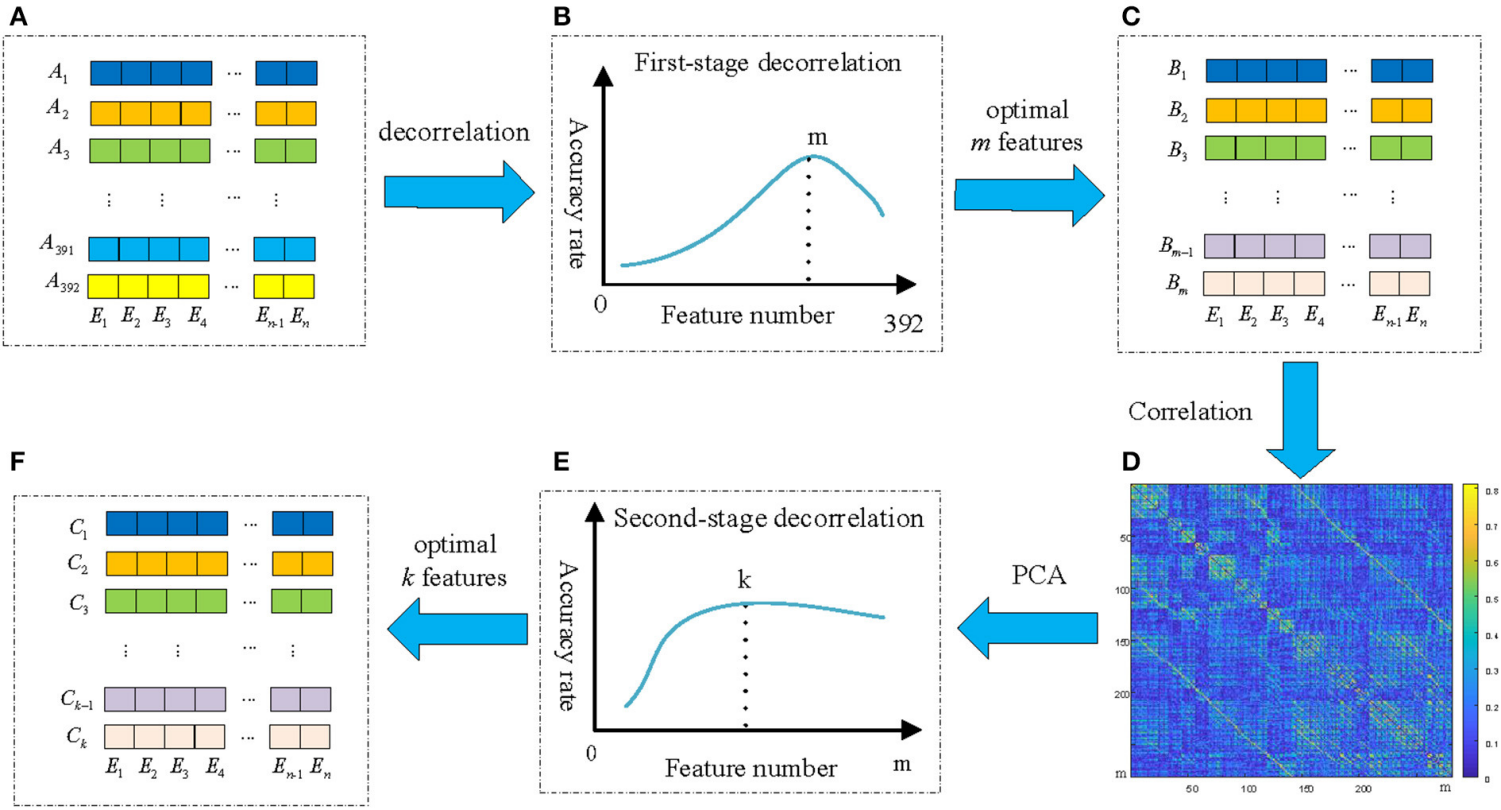
The framework of the proposed ATD feature extraction method. **(A)** The original 392 *n-dim* feature vectors; **(B)** The emotion recognition performance of the first-stage decorrelation; **(C)** Selected *m* optimal feature vectors by first-stage decorrelation; **(D)** The correlation coefficient matrix of *m* feature vectors; **(E)** The emotion recognition performance of the second-stage decorrelation; **(F)** Selected *k* optimal feature vectors by second-stage decorrelation.

It is obviously that there are many highly correlated redundant features among the 392 feature vectors, and the correlation coefficient matrix is symmetric. If these redundant feature vectors can be removed, the effectiveness of the features can be improved. In addition, since the GC brain network of different EEG signals are different, and redundancy connections in the corresponding brain network are also different. Therefore, the redundancy method should be able to adapt to the variation of different EEG signals. In this paper, we propose a new feature extraction and optimization method of ATD method to meet the requirements, and the framework of the proposed ATD method is shown in Figure 7.

**Figure 8:**
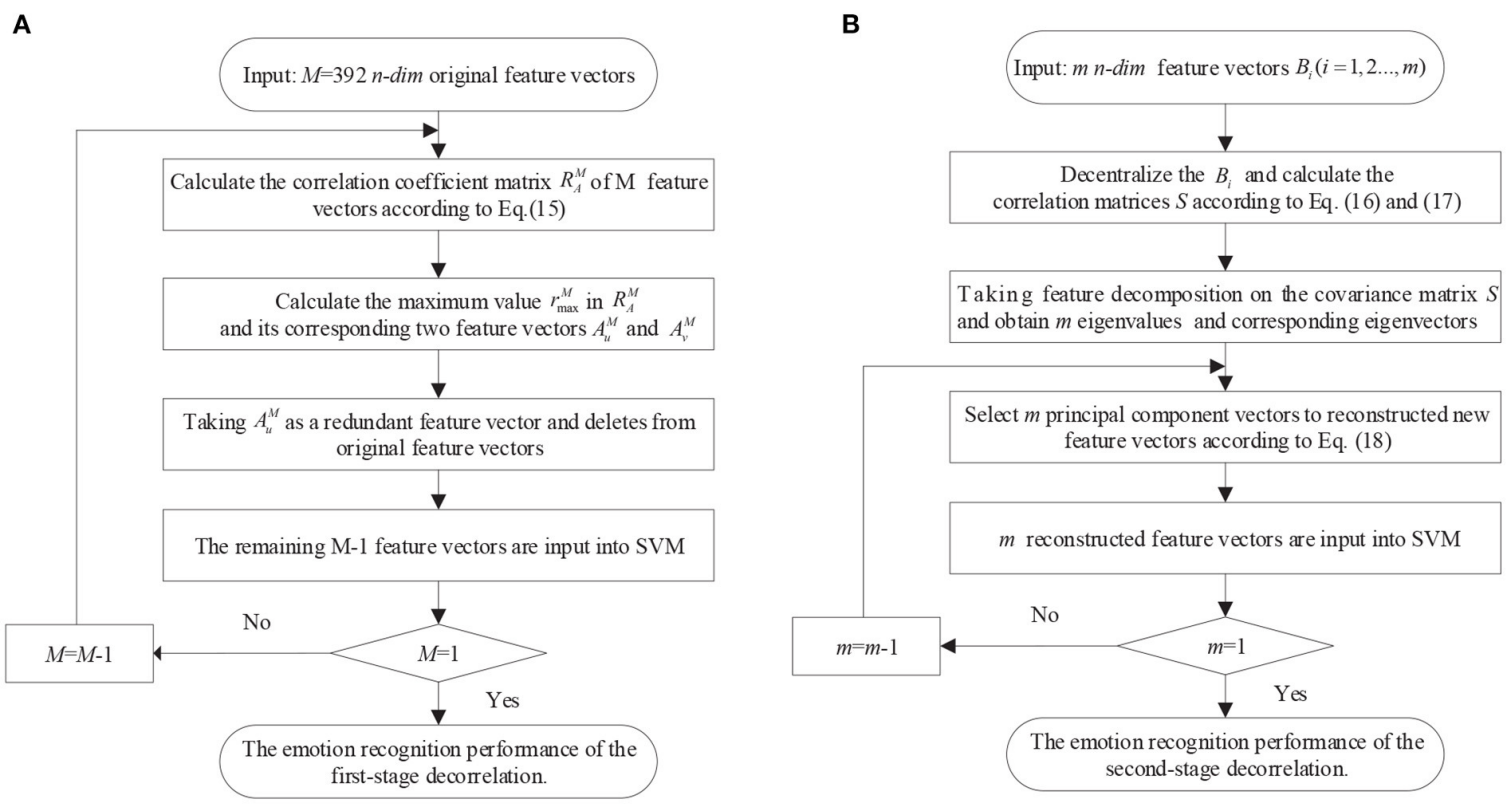
The framework of the ATD method. **(A)** The framework of the first-stage decorrelation; **(B)** The second-stage decorrelation.

The first-stage decorrelation of the original 392 *n-dim* feature vectors is shown in Figure 8A. Here M is used to represent the number of the feature vectors and initialized to 392. Corresponding to the above-mentioned symbols, we add the superscript M to all them, such as *R*_*A*_ rewritten as $R_{A}^{M}$. Also, due to the symmetry of $R_{A}^{M}$, this paper only selected the lower triangular matrix for correlation analysis.

First of all, the maximum value $r_{\max}^{M}$ in the correlation coefficient matrix $R_{A}^{M}$ and its corresponding two feature vectors $A_{u}^{M}$ and $A_{v}^{M}$ were determined, and randomly select one of these two vectors as a redundant vector and remove from the original feature vectors. In this paper, we compared four kinds of redundant vectors definition methods: (1) the first feature vector $A_{u}^{M}$ is selected as redundant vector; (2) the second feature vector $A_{v}^{M}$ is selected as redundant vector; (3) randomly selects one of them as redundant; (4) Calculate the correlation between the $A_{u}^{M}$ and $A_{v}^{M}$ and the remain M-2 feature vectors, and the highest correlation feature vector is selected as redundant. The experimental results show that the emotion recognition performance obtained by the above four methods is almost the same. Therefore, we adopt the simplest method, that is, the first feature vector $A_{u}^{M}$ is deleted as of a redundant feature vector, and the remaining *M*−1 feature vectors are used as the input of the SVM classifier and obtained the recognition accuracy. Then, the above process is repeated for the remaining M-1 feature vectors until the number of remaining features is 1. Figure 7B shows the relationship of emotion recognition accuracy with the different number of feature vectors. It can be seen that there is an optimal number of *m*(*m* <392) to make the emotion recognition performance reach the highest and retains these *m* feature vectors *B*_*i*_(*i* = 1, 2, …, *m*) as optimal feature vectors of the first-stage decorrelation features, as presented in Figure 7C.

To further explore the correlation of the above *m* features vectors, the corresponding correlation matrices $R_{B}\in {\mathbb{R}}^{m*m}$ between *B*_*i*_ are calculated according to Equation (15) and shown in Figure 7D. We can find that there is still a certain correlation between these feature vectors, and it is necessary to furtherly remove the correlation between them. As we all know, Principal components analysis (PCA) (Li et al., 2016) is a statistical procedure that uses an orthogonal transformation to convert a set of correlated variables into a set of values of linearly uncorrelated variables, and the main idea of PCA is to reduce the number of variables while preserving as much information as possible. Therefore, we then adopt the PCA method to secondly remove the correlation of the *B*_*i*_, and the detailed process is shown in Figure 8B.

First, *m* feature vectors *B*_*i*_(*i* = 1, 2…, *m*) are decentralized, that is, the mean values of *m* feature vectors are subtracted from each other to ensure that the mean value of each feature vector is 0. The *m* decentralized feature vectors *B*_*M*_ can be represented as:


(16) $$\begin{array}{l} B_{M}=B_{i}-\bar{B_{i}}\left(i=1,2\ldots ,m\right) \end{array}$$

Then, the correlation matrices *S* ∈ ℝ^m*m^ of *m* feature vectors *B*_*M*_ is calculated as follows:


(17) $$\begin{array}{l} S=\frac{1}{n-1}B_{M}B_{M}^{T} \end{array}$$

After this, we perform feature decomposition on the covariance matrix *S* and obtain *m* eigenvalues λ_1_, λ_2_…, λ_*m*_ and corresponding eigenvectors ξ_1_, ξ_2_, …ξ_*m*_. Taking the first p eigenvalues and the corresponding eigenvectors to construct the orthogonal matrix *V* = (ξ_1_, ξ_2_, …ξ_*p*_), where each column in *V* corresponds to a principal component. Based on this, the reconstructed feature vectors *B*′ with the size of *p*×*n* are obtained, as described in Equation (18), which are input into the SVM classifier to obtain the corresponding emotion recognition accuracy.


(18) $$\begin{array}{l} B^{\prime }=V^{T}B \end{array}$$

Finally, different values *p(p* < *m)* were selected to repeat the above process, and the performance of the second-stage decorrelation method with the different number of feature vectors was obtained and shown in Figure 7E. It can be seen that there exists an optimal number of feature vectors *k*(*k*<*m*) to optimize the emotion recognition performance, and the *k* feature vectors *C*_*i*_(*i* = 1, 2…, *k*) are retained, as shown in Figure 7F.

Above all, we can get the GC+ATD features after removing redundant connections in the GC correlation matrix by ATD method, which will be used for EEG emotion recognition. Because the redundant connections in each GC brain network are always different, the correlation degree of the redundant connections is also different. Therefore, it is necessary to adaptively select the optimal *m* and *k* in the above ATD process to ensure the extracted causal features can achieve the best recognition performance.

### 3.3. The proposed GC+ATD multi-feature weighted fusion method

According to whether the frequency domain and hemispheres domain are used, GC analysis of EEG signals is divided into four categories, as shown in Table 1. A single type of GC feature can only show a part of the causality information. Therefore, it is necessary to integrate four GC+ATD features to make full use of the causal complementarity among them.

Most of the existing research on feature fusion method is commonly used in series, or in parallel or weighted superpositions. Generally speaking, if the number of features is large and the structure is different, the fusion algorithm is complex and the computational complexity is higher. In addition, the four GC+ATD features in this paper have structural similarities. Therefore, considering the computational complexity and performance, a new multi-feature weighted fusion scheme is proposed to balance the recognition rate and feature numbers, which designing the weight function for each GC+ATD feature by comprehensively considering the recognition rate and the feature number of each single feature.

Let *T*_*i*_ and *T*_*final*_ represent the *i-th* GC+ATD feature and the final fusion feature, respectively. The problem of the proposed feature fusion scheme can be expressed as:


(19) $$\begin{array}{l} T_{final}=\overset{4}{\underset{i=1}{\sum }}w_{i}T_{i} \end{array}$$

where *w*_*i*_ is the weight of *T*_*i*_, it can be calculated as follows.

Let *R*_*i*_ is the recognition accuracy of *T*_*i*_. *N*_*i*_ and *N*_*all*_ represent the feature number of *T*_*i*_ and *T*_*final*_, respectively. To ensure the features *T*_*i*_ with better recognition performance and smaller feature numbers have a higher importance in *T*_*final*_, the weight coefficients *w*_*i*_ are designed as follows.


(20) $$\begin{array}{l} w_{i}=p_{i}R_{i}+q_{i}\left(1-\frac{F_{i}}{F_{all}}\right)\left(i=1,2,3,4\right) \end{array}$$

where *p*_*i*_ and *q*_*i*_ represent the importance of recognition accuracy and feature number of the *T*_*i*_, and *p*_*i*_+*q*_*i*_ = 1.

In summary, to maximize the emotion recognition accuracy *R*_*T*_*final*__ of the fusion GC features *T*_*final*_, the problem of the above multi-feature fusion scheme can be expressed as follows:


(21) $$\begin{array}{l} \begin{array}{l} \underset{p_{i}\in \left[0,1\right]}{\text{arg max }}R_{T_{final}}\\ s.t.T_{final}=\overset{4}{\underset{i=1}{\sum }}w_{i}T_{i}\\ {\;w}_{i}=p_{i}R_{i}+\left(1-p_{i}\right)\left(1-\frac{F_{i}}{T_{all}}\right) \end{array} \end{array}$$

Since the objective function in Equation (21) is not convex, and there is only one unknown parameter *p*_*i*_, we use an exhaustive search to find the optimal *p*_*i*_ with the search range of [0, 1] and search step of 0.05. The final four optimal *w*_*i*_ are used for multi-GC feature fusion. It can be seen from Equation (21) that the higher the recognition accuracy and the less the number of one feature, the final weight *w* will be larger, which means the higher the importance in the final decision fusion method will be.

## 4. Experimental results and discussion

### 4.1. Experimental Settings

In this paper, we conducted three groups of experiments to evaluate the performance of the proposed scheme. In the first group, the proposed four kinds of GC measures are evaluated. Next, we test the performance of the ATD+GC feature extraction scheme. Third, we give some experimental results to discuss the performance of the proposed multi-GC feature fusion scheme. All the experiments are carried out in the same environment, parameter settings, and evaluation indexes. The hardware environment is a Dell XPS 8930 desktop computer with the CPU is Intel Core i7-8700K@3.70GHz and 16GB memory, and the software environment is MATLAB2019b. Since the main study of this paper is on the effectiveness of feature extraction rather than the recognition model, and we adopt the most widely used SVM classifier for all the emotion recognition processes.

**Figure 9:**
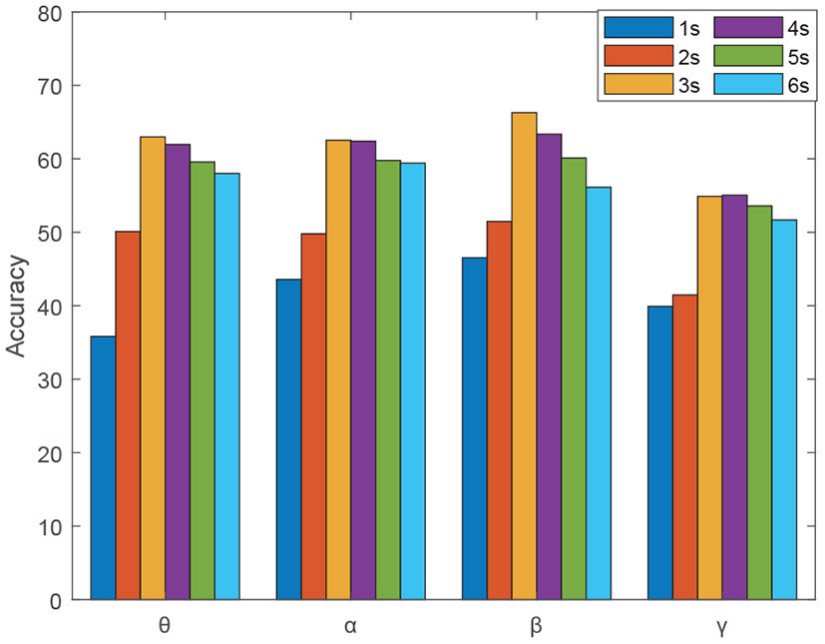
The effect of the time window size on the EEG emotion recognition.

All the experimental results in this paper were tested on the DEAP dataset. After the preprocessing in Section 2.2, we segmented each trial EEG signals of each subject. To further validate the effectiveness of our method, we investigated the effect of the time window size on the EEG emotion recognition, where the EEG signals are segmented into 1, 2, 3, 4, 5, and 6 s. As seen from the results in Figure 9, the GC features of different frequency bands both showed sensitivity to the time window size and the time window size of 3 s shows the best recognition performance. Otherwise, the study in Li et al. (2017) and Tao et al. (2020) also analyzed the emotion classification with DEAP EEG signals from the perspective of time window size and obtained the highest accuracy of 3s signal segments, which is similar to the results we obtained.

Therefore, we segmented each 1-min trial into 39 segments with a window length of 3s and an overlap time of 1.5 s, and the 40 trial EEG signals of each subject are divided into 1560 EEG samples. Finally, the training data and test data are divided by 8:2, and the average recognition accuracy by 5-fold cross-validation of 32 subjects is calculated to evaluate the performance of the proposed scheme.

### 4.2. The emotion recognition performance of the proposed GC measures

This section mainly tests the performance of the proposed GC measure, and the GC values of the adjacency matrices are directly used as features for emotion recognition. Table 2 show the EEG emotion recognition performance of the SS, SC, CS and CC measures.

**Table 2:** The recognition performance of the proposed SS, SC, CS, and CC measures (%).

**GC Measures**		**HAHV**	**HALV**	**LAHV**	**LALV**	**Average**
SS	θ~θ	75.54	70.18	43.14	63.08	62.98
	α~α	73.18	74.38	40.98	61.54	62.52
	β~β	80.08	73.67	47.04	64.36	66.29
	γ~γ	68.64	69.24	22.21	59.49	54.89
CS	θ~θ	68.61	63.52	28.72	56.07	54.23
	α~α	66.25	62.79	27.09	53.11	52.31
	β~β	68.47	64.09	25.31	57.78	53.91
	γ~γ	69.84	54.25	22.00	37.75	45.96
SC	θ~α	76.40	70.83	38.44	64.06	62.43
	θ~β	65.99	53.86	5.87	26.82	38.14
	θ~γ	62.43	43.64	14.73	29.99	37.70
	α~β	69.66	66.06	52.64	53.96	60.58
	α~γ	64.84	56.54	25.31	45.86	48.14
	β~γ	78.49	79.95	62.79	72.05	73.32
CC	θ~α	72.00	65.05	38.01	61.54	59.15
	θ~β	63.37	56.98	26.09	35.90	45.58
	θ~γ	58.20	51.31	17.50	39.49	41.62
	α~β	76.93	69.23	38.88	61.80	61.71
	α~γ	62.14	52.45	22.66	38.72	43.99
	β~γ	74.51	80.23	53.19	69.23	69.29
SS (4)		79.88	70.25	77.40	53.39	70.23
CS (4)		76.09	68.28	45.64	57.19	61.80
SC (3)		85.27	82.35	58.44	72.18	74.56
CC (3)		85.01	73.85	80.89	61.53	75.32

It can be seen that the proposed four GC measures can achieve better recognition performance in Table 2. For the SC and CC measures with the cross-frequency bands, the adjacent frequencies, such as θ~α, α~β, and β~γ measures can achieve better emotion recognition performance, and the SC(β~γ) and CC(β~γ) have the highest recognition accuracy of 73.32 and 69.29%, respectively. However, the non-adjacent frequencies, such as θ~β, θ~γ, and α~γ, which show poor recognition performance. This further quantitatively verify the conclusions in Figure 5 in Section 3.1.2. Therefore, the non-adjacent frequency GC measures will no longer be considered in the following section.

Moreover, Table 2 also presents the emotion recognition performance of the direct cascade combinations of different GC measures, where SS(4), CS(4), SC(3), and CC(3) represent the concatenated combinations of the four SS measures, four CS measures, three SC measures, and three CC measures, respectively. The experimental results show that, compared with a single feature, the recognition performance of combination features was significantly improved. This proves that the causal relationship of different combinations of the same GC measure is complementary. Additionally, the cross-frequency measures SC(3) and CC(3) can achieve an average of 8.55% and 9.30% improvement compared with the same-frequency measures SS(4) and CS(4), respectively. It is further proof that there is a signification causal relationship between EEG signals with cross-frequency bands in the left and right hemispheres.

### 4.3. The performance of the proposed ATD method

Since the four GC adjacency matrices have similar structures as shown in Figure 6, this section takes CC measure as an example to evaluate the performance of the proposed ATD method (GC+ATD). Four reference schemes were selected: GC+PCA (Li et al., 2016), GC+Decorrelation (Weinstein et al., 1993), GC+K-means (Orhan et al., 2011), and GC+the Restricted Boltzmann Machine(RBM) (Hinton and Salakhutdinov, 2006). Table 3 shows the emotion recognition performance.

**Table 3:** The recognition performance of different CC feature combinations.

**Feature**	**Accuracy (%)**			**Feature numbers**			**Run time**		
	**(θ~α)**	**(α~β)**	**(β~γ)**	**(θ~α)**	**(α~β)**	**(β~γ)**	**(θ~α)**	**(α~β)**	**(β~γ)**
GC	59.15	61.71	69.29	392	392	392	542	546	544
GC+PCA	53.17	58.53	69.24	80	130	85	553	558	554
GC+Decorrelation	59.03	59.70	70.91	242	340	290	576	594	578
GC+K-Means	56.86	56.86	56.86	330	270	350	635	641	633
GC+RBM	51.43	51.07	59.72	100	100	100	578	583	580
GC+ATD	61.39	64.63	71.15	71	83	77	596	605	590

Judging from the results, the proposed GC+ATD can achieve an improvement of 2.24, 2.92, and 1.86% than three CC adjacency matrix features, respectively. Compared with the GC+PCA, GC+Decorrelation, GC+K-means, and GC+RBM, GC+ATD is always better with average improvements of 5.41, 2.51, 8.87, and 11.65%, and the average running time increased by 7.57, 2.46, –6.18, and 2.87%, respectively. The experimental results show that the GC+ATD can effectively improve the performance of emotion recognition with less extra time complexity. In addition, Table 3 also shows the feature numbers of different methods, where the feature numbers of GC+ATD is the smallest in most cases.

### 4.4. Performance of the proposed multi-GC feature fusion strategy

In this section, we evaluate the performance of the proposed GC+ATD multi-feature weighted fusion scheme, the emotion recognition performance is shown in Table 4. In order to verify the effectiveness of the proposed scheme, the direct cascading feature scheme is taken into comparison. The following discussion can be obtained from the results:

1. Compared with the single GC feature, GC+ATD features can achieve an average improvement of 0.85%, the running time of the model increased by 10.52%, and the average number of features decreased by 87.07% of the GC features. This means that the ATD method can effectively reduce the number of features without reducing the performance of emotion recognition and less extra time complexity.
2. For the same-frequency fusion features of SS+CS and the cross-frequency fusion features of SC+CC, the GC+ATD feature can achieve an improvement of 0.54 and 2.33% than that of GC features, the running time of the model increased by 11.48 and 10.40%, and the average number of features both decreased by 87.07% of the GC features. Otherwise, the two kinds of fusion features have the same feature numbers, but the emotion recognition performance of cross-frequency fusion features is 8.72% higher than that of same-frequency fusion features. This indicates that there is a significant difference in emotional EEG signals between the cross-frequency bands.
3. For the same-hemisphere fusion features of SS+SC and the cross-hemisphere fusion features CS+CC, the GC+ATD feature can achieve an improvement of 1.15 and 6.16% than that of GC features, the running time of the model increased by 12.22 and 10.25%, and the average number of features decreased by 86.88 and 87.35% of the GC features, respectively. Compared with the same-hemisphere fusion features, the emotion recognition of the cross-hemisphere fusion features increased by 2.20 and the number of features decreased by 31.11%, indicating that the emotion EEG signals have a more obvious causal difference in the cross-hemisphere.
4. For the four features fusion with the direct cascade fusion method and the proposed multi-feature fusion method, the GC+ATD feature can achieve an average improvement of 3.06 and 8.42% than the GC feature, the running time of the model increased by 11.38 and 11.43%, and the average number of features both decreased by 87.07% of the GC features. Otherwise, the emotion recognition accuracy of the proposed GC+ATD multi-feature weighted fusion method reaches 84.91%, which is 5.36% higher than that of the direct cascade fusion method. The results further verify that both recognition accuracy and the feature number of a single feature can effectively improve the performance of EEG emotion recognition.
5. Comparing the single feature, two fusion features, and four fusion features, the GC+ATD feature always has the best emotion recognition performance. Among them, the emotional recognition performance of four fusion features is 11.42 and 11% higher than that of a single feature and two fusion features. The results further verify that both recognition accuracy and the computational complexity of a single GC feature affect the performance of the feature fusion scheme, and they can interact to improve the final emotion recognition performance.

**Table 4:** The GC value numbers of different measures.

**Feature**		**Accuracy (%)**		**Feature number**		**Run time(s)**	
		**GC**	**GC+ATD**	**GC**	**GC+ATD**	**GC**	**GC+ATD**
Single	SS	70.23	70.21	1,568	180	2,167	2,420
	CS	61.80	63.34	784	124	1,081	1,170
	SC	74.56	75.56	1,176	180	1,638	1,811
	CC	75.32	76.21	1,176	124	1,632	1,803
Two	SS+CS	69.01	69.55	2,352	304	3,248	3621
	SC+CC	75.94	78.27	2,352	304	3,270	3,640
	SS+SC	73.13	74.28	2,744	360	3,805	4,270
	CS+CC	70.51	76.67	1,960	248	2,713	2,991
Four	Cascade	76.49	79.55	4,704	608	6,518	7,260
	Proposed	76.49	84.91	4,704	608	6,518	7,263

To further verify the effectiveness of the proposed method, we will compare the proposed scheme with several state-of-the-art reference features for the DEAP dataset. several reference features are chosen: the asymmetry features of the left and right hemispheres such as DASM and RASM (Zheng and Lu, 2015), the asymmetry features of the differential caudality (DCAU) (Zheng and Lu, 2015), and the traditional GC feature (Gao et al., 2020). All the schemes adopt the same EEG signal division and 5-fold cross-validation with SVM classifiers, and the recognition accuracy of each scheme is shown in Table 5. The emotion recognition accuracy of the proposed GC+ATD scheme can reach 84.91%, which is always better than DASM+RASM and DACU features with average improvements of 15.24% and 17.49%, respectively. Compared with the transfer entropy (TE) (Shi et al., 2018) and phase amplitude coupling (PAC) (Jin et al., 2022) features of EEG signal in the same-frequency bands and cross-frequency bands, the proposed ATD-GC feature can achieve an average improvement of 10.37 and 13.35%, respectively. In addition, compared with the traditional GC feature with the same-frequency band, the GC+ATD method has increased by 8.43%, this result further proves that the cross-frequency causal analysis of EEG signals can further improve the performance of emotion recognition.

**Table 5:** The recognition performance with latest literatures (%).

**Method**	**Feature**	**Classifier**	**Classes**	**Accuracy**
Zheng and Lu (2015)	DASM,RASM	SVM	4	69.67
Zheng and Lu (2015)	DCAU	SVM	4	67.42
Shi et al. (2018)	Transfer entropy	SVM	4	74.54
Jin et al. (2022)	Phase amplitude coupling	SVM	4	71.65
Gao et al. (2020)	GC	SVM	4	76.48
This paper	GC+ATD	SVM	4	84.91

## 5. Conclusion

In this paper, combining the asymmetry of the left and right hemispheres and the GC relationship of the EEG signals, an emotion recognition scheme based on multi-GC feature extraction and fusion in the left and right hemispheres is proposed. First of all, the GC relationship of the EEG signals is divided into four categories according to whether the EEG signals belong to the same hemisphere and frequency. we mainly analyze the GC relationship of EEG signals with the cross-frequency band, making the causal analysis of the EEG signal more compliable. Then, we design an ATD feature extraction method to adaptive remove the redundant connections in the GC brain network, which can effectively reduce the number of features without reducing the emotion recognition performance. Finally, considering the recognition accuracy and the computational complexity of each single GC feature, a new multi-feature weighted fusion scheme is designed, which pays closer attention to the GC feature with higher recognition accuracy and lower feature numbers during the fusion process. The results on the DEAP emotion dataset show the GC+ATD features can achieve an improvement of 8.43% than the GC feature, and the proposed multi-feature weighted fusion scheme is 5.36% higher than that of the direct cascade fusion method. The results of this paper show that it is necessary to take the cross-frequency causality of the EEG signal as part of causal attributes to enhance the causality of EEG signals and improve the performance of emotion recognition.

## Data availability statement

The original contributions presented in the study are included in the article/supplementary material, further inquiries can be directed to the corresponding author/s.

## Ethics statement

Written informed consent was obtained from the individual(s) for the publication of any potentially identifiable images or data included in this article. Ethical review and approval was not required for the study on human participants in accordance with the local legislation and institutional requirements.

## Author contributions

JZ and XZ contributed to the conception and design of the study. JZ organized the database, performed the analysis, and wrote the first draft of the manuscript with the support of XZ and GC. GC, LH, and YS contributed to the manuscript revision. All authors participated to the scientific discussion. All authors contributed to the article and approved the submitted version.

## Funding

This work was supported by Shanxi Scholarship Council of China (HGKY2019025 and 2022-072).

## Conflict of interest

The authors declare that the research was conducted in the absence of any commercial or financial relationships that could be construed as a potential conflict of interest.

## Publisher's note

All claims expressed in this article are solely those of the authors and do not necessarily represent those of their affiliated organizations, or those of the publisher, the editors and the reviewers. Any product that may be evaluated in this article, or claim that may be made by its manufacturer, is not guaranteed or endorsed by the publisher.
